# Development and Validation of Low-Cost Indoor Air Quality Monitoring System for Swine Buildings

**DOI:** 10.3390/s24113468

**Published:** 2024-05-28

**Authors:** Elanchezhian Arulmozhi, Anil Bhujel, Nibas Chandra Deb, Niraj Tamrakar, Myeong Yong Kang, Junghoo Kook, Dae Yeong Kang, Eun Wan Seo, Hyeon Tae Kim

**Affiliations:** 1 Department of Bio-Systems Engineering, Institute of Smart Farm, Gyeongsang National University, Jinju 52828, Republic of Korea; mohanachezhian@yahoo.com (E.A.);; 2 Ministry of Communication and Information Technology, Singhadurbar, Kathmandu 44600, Nepal; 3 Department of Smart Farm, Institute of Smart Farm, Gyeongsang National University, Jinju 52828, Republic of Korea

**Keywords:** air quality, Raspberry Pi, IoT, sensor networks, swine building

## Abstract

The optimal indoor environment is associated with comfortable temperatures along with favorable indoor air quality. One of the air pollutants, particulate matter (PM), is potentially harmful to animals and humans. Most farms have monitoring systems to identify other hazardous gases rather than PM due to the sensor cost. In recent decades, the application of environmental monitoring systems based on Internet of Things (IoT) devices that incorporate low-cost sensors has elevated extensively. The current study develops a low-cost air quality monitoring system for swine buildings based on Raspberry Pi single-board computers along with a sensor array. The system collects data using 11 types of environmental variables along with temperature, humidity, CO_2_, light, pressure, and different types of gases, namely PM_1_, PM_2.5_, and PM_10_. The system is designed with a central web server that provides real-time data visualization and data availability through the Internet. It was tested in actual pig barns to ensure stability and functionality. In addition, there was a collocation test conducted by placing the system in two different pig barns to validate the sensor data. The Wilcoxon rank sum test demonstrates that there are no significant differences between the two sensor datasets, as all variables have a p-value greater than 0.05. However, except for carbon monoxide (CO), none of the variables exhibit correlation exceeding 0.5 with PM concentrations. Overall, a scalable, portable, non-complex, low-cost air quality monitoring system was successfully developed within a cost of USD 94.

## 1. Introduction

Given the growing global population and the quest for improved nutrition, it is crucial to acknowledge that pork production holds unparalleled importance as a primary source of protein in numerous countries. Pork is globally the second most consumed meat after poultry and beef, which account for 92% of total meat produced [1,2]. Pigs are commonly raised in enclosed pig barns, closed pig barns, or confinement buildings. These structures protect pigs from harsh weather conditions and facilitate better management of pigs, including monitoring, feeding, vaccination, and waste handling [3]. The optimal indoor environment is associated with good indoor air quality and comfortable temperatures. In contrast, pollutants accumulating within confinement facilities can harm both human beings and animals housed in them. One environmental pollutant, particulate matter (PM), consists of solid particles and liquid droplets suspended in the atmosphere. High concentrations of PM, mainly delicate particulate matter, which can penetrate the alveoli during respiration due to their size, pose a potential threat to worker safety and animal welfare [4]. Scientists categorize PM into PM_10_ (inhalable particles with diameters generally 10 µm (10 μm) and smaller), PM_2.5_ (fine inhalable particles with diameters of generally 2.5 µm and smaller), and PM_1_ (extremely fine particles with a diameter less than 1 µm, significantly smaller than PM_2.5_). PM_2.5_ is a significant global public health concern, notorious for having various detrimental effects on cardiopulmonary health [5]. Its adverse impacts extend to the male reproductive system upon exposure. Research indicates that PM_10_ significantly affects the respiratory health of finishing pigs, with nursery pigs demonstrating greater sensitivity to particulate matter than fattening pigs due to their comparatively weaker immune systems [3]. Despite the comprehensive documentation of the health effects associated with PM, no identified threshold concentration level for PM guarantees the absence of adverse health effects. While monitoring PM concentration is crucial for assessing air quality; swine farmers often overlook this measure due to the perceived expense of the monitoring methods.

Past research emphasizes the importance of real-time indoor air quality monitoring, as understanding the air quality within confinement buildings is crucial for evaluating potential risks [5]. Typically, evaluating indoor air quality in confinement buildings may require the services of professional environmental testing equipment, such as TEOM (Model 1400 AB, Thermo Scientific, Waltham, MA, USA), DustTrak (Model 8520, TSI Inc., Shoreview, MN, USA), HAZ-DUST (Model EPAM-5000, SKC Inc., Fullerton, CA, USA), and others [6]. While choosing professional equipment, they are impressive in measuring air quality in pig barns. Unfortunately, there are certain disadvantages to acquiring such equipment, which are detailed as follows. **Cost:** Professional-grade equipment is often expensive, making it a significant investment for farmers, especially smaller operations; **Complexity:** These instruments can be complex to operate and may require trained personnel, which can add to operational costs and the need for specialized training; **Maintenance:** Regular maintenance is essential to ensure accurate and reliable measurements. This can be time-consuming and may require technical expertise; **Limited Accessibility:** Due to the high cost, not all farmers may have access to such equipment, limiting widespread adoption and monitoring in smaller or resource-constrained farms; **Real-time Data Challenges:** Some professional equipment may not provide real-time data, leading to delays in identifying and responding to changes in air quality; **Specificity:** Some equipment may be designed for specific pollutants and using them for a broader range of air quality parameters may not be as accurate or comprehensive; **Portability:** Some professional instruments may lack portability, making it challenging to monitor air quality in the different areas of a pig barn; **Limited Sensor Capabilities:** Some professional air quality sensors may be designed specifically for particulate matter or certain gases, and they might not be equipped to measure additional parameters like temperature, humidity, light, sound, motion, vibration, flame, or other types of gases; **Sensor Integration Challenges:** Integrating multiple sensors for different parameters may be challenging, especially if the sensors come from different manufacturers. Compatibility issues can arise regarding communication protocols, data formats, and power requirements; **Calibration:** Regular calibration is necessary to maintain accuracy. Calibration processes can be intricate and require technical knowledge; **Power Requirements:** Certain professional instruments may have specific power requirements, making them less suitable for locations with limited power infrastructure.

To overcome this challenge, there is a demand for environment monitoring systems that are affordable, scalable, and easily accessible. The current phase of agriculture, known as agricultural era 4.0, incorporates modern technologies like the Internet of Things (IoT), big data analysis, artificial intelligence, cloud computing, and remote sensing [7,8,9]. The IoT has played a pivotal role in various sectors, including industry, healthcare, agriculture, logistics, transportation, power grids, environmental protection, fire safety, furniture, and other facets closely intertwined with daily life. Incorporating these cutting-edge technologies has significantly improved agricultural practices by developing single-board computers such as Raspberry Pi, which are compatible with cost-efficient sensors and open network platforms. Advancements in information technology, sensor technology, and IoT technology have made it feasible to construct a comprehensive system that is well suited for continuously monitoring the environment of livestock buildings over an extended period. In recent decades, the application of environmental monitoring systems based on IoT devices that incorporate low-cost sensors has significantly increased. Along with the low-cost sensor technology, the availability of open source furthered the proliferation of IoT-based environmental monitoring. In a broad sense, the term “open source” pertains to software applications where the source code is accessible to the public so they can modify or utilize the program as intended.

This research paper explores developing and implementing a low-cost Raspberry Pi-based air quality monitoring system tailored to the specific needs of livestock management. We will discuss the hardware and sensor components, the data collection and visualization techniques, and the potential benefits of such a system for livestock health and farm productivity. Furthermore, it has undergone comprehensive testing and validation in an actual environment and was assessed through co-locating testing. This research endeavors to make a valuable contribution to the welfare of livestock, farmers’ health, and the agricultural industry’s sustainable development by offering a cost-effective and easily accessible air quality monitoring solution for livestock buildings. The subsequent sections of this paper are organized in the following manner. The experimental site and setup are described in Section 2, which focuses on materials and methods. Section 3 of the document outlines the prototype’s system architecture, along with detailed descriptions of the software and hardware components. In Section 4, an examination is conducted on the outcomes of the co-location test and the system’s performance. Section 5 and Section 6 are dedicated to the discussion and conclusion of the respective sections.

### Literature Review

While data collection has always been difficult and costly in smart building research, it is essential for the creation of smart agricultural buildings. To address this, IoT-based monitoring systems have been put into practice. However, the ability to acquire, gather, and process continuous indoor air quality data in agricultural buildings at a reasonable cost is still limited. In indoor environmental monitoring, several earlier studies are being put forth and making substantial contributions; these will be covered in the current part.

A previous study [10] did a laboratory assessment of low-cost PM monitors to evaluate the performance of four low-cost PM monitors (Speck (Airviz Inc., Pittsburgh, PA, USA), Dylos 1100 Pro/Dylos 1700 (Riverside, CA, USA), AirAssure PM_2.5_ IAQ Monitor (TSI Inc., Shoreview, MN, USA), and AirSense (Buffalo, NY, USA)) against well-characterized reference instruments. Even though a critical assessment was conducted between multiple sensors, those are not custom-built, and the data collection system is poor, such as stored in the SD card or 9-pin serial cable. Likewise, Ref. [11] evaluated the performance of low-cost sensors for air quality measurements (PM_1_, PM_2.5_, and PM_10_). They have used GRIMM Aerosol (GRIMM EDM 107) and plantower (pms5003) to collect the data as a collection test. However, the major objective of this study is to compare both sensors with public data monitoring rather than building a database management system. Additionally, Ref. [12] accessed three different sensor units from AQMes (AQMesh pods (Environmental Instruments Ltd., Stratford-upon-Avon, UK) with a colocation test. That study asses the NO_2_, NO, O_3_, temperature (T), relative humidity (RH) and atmospheric pressure (P). That study evaluated the performance during the winter and summer seasons of Parma and Modena provinces of northern Italy. However, it is imperative for an effective data collection system to offer up-to-date air quality data, which can be valuable in comprehending the surrounding environment and aiding decision makers in formulating more effective pollution control policies. The utilization of a low-cost air quality monitoring system powered by a single-board computer (SCB) for the measurement of indoor air pollutants has experienced a significant increase in popularity in recent years [13]. 

A wireless sensor network system combined with the XBee module, Arduino, and Raspberry Pi as open source hardware platforms was proposed by a previous study [13]. The system is inexpensive and expandable in terms of the kind of sensors and the number of sensor nodes. An Arduino board was intercropped with several sensors acting as the data acquisition unit/sensing nodes. The prototype was designed to gather data on humidity and temperature. An XBee module integrated with Raspberry Pi Zero is used to create an access point that allows a continuous flow of data from the Raspberry Pi Zero to the data storage layer. The base station is built around a Raspberry Pi, which is connected to the Internet via a router. The base station’s XBee module functions as a coordinator device and can only support a maximum of 10 sensing nodes. The system is portable in the sense that the sensing nodes can be placed in proximity to the base station. It is appropriate for a broad range of environmental monitoring applications. In a previous study [14], an Android-based smart home system was developed that can be controlled via Bluetooth and Internet connectivity. An Arduino Ethernet microweb server running consists of Arduino Mega 2560 and Arduino Ethernet Shield used as the motherboard of the system, which consists of a siren, a nRF24L01+ radio module to control the devices such as light switches, temperature sensors, gas sensors, motion detection sensors, and alarms in a household. 

However, several studies were developed to control and automate residential buildings and office buildings. For instance, Ref. [15] developed indoor air quality monitoring for multi-point based on IoT to monitor the air quality and microclimate of a residential building. That study utilizes a custom-made motherboard and resistance–capacitance (RC) elements to build the monitoring system, along with three sensors to monitor the temperature, humidity, CO_2_, and PM_2.5_. The distribution monitoring page, historical data report, and monitoring alarm functions were added to their own designed web page, along with the data visualization of collected variables using Modbus protocol. Likewise, another recent study [16] implemented an indoor environment monitoring system for residential buildings, a Raspberry Pi-based multi-sensor microclimate monitoring system. They used multiple sensors, including a temperature and humidity sensor (DHT11), a light sensor (GL5528), a microphone sound sensor, flame sensor, a vibration sensor (SW420), and a passive infrared motion sensor (HC-SR501) to collect environmental variables. They also conducted a case study to check the performance of the system in different rooms in that house. The systems suggested in Reference [16] possess the characteristic of being cost-effective, portable, and capable of scaling to a certain degree. 

Nonetheless, the implementation of IoT systems is imperative in sectors such as agriculture and livestock management, which are susceptible to current climatic conditions but are not conducive to ensuring food security. While there have been many studies on the use of IoT in agriculture [7,17,18,19,20,21] there is a noticeable lack of research on the air quality monitoring systems specifically in livestock buildings. The current study aims to address these limitations by focusing on the development of a cost-effective, straightforward, effective, scalable, and portable indoor air quality monitoring system utilizing Raspberry Pi technology. To ensure cost-effectiveness, the study employed inexpensive sensors, while wireless communication was utilized to achieve portability. 

## 2. Materials and Methods

### Site Description

**Figure 1:**
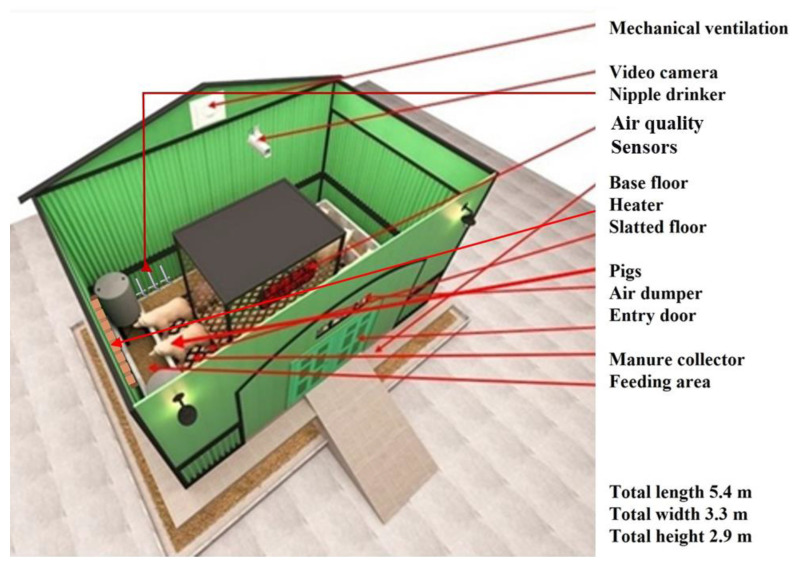
The experimental site with dimensions and sensor placements.

The present study was conducted in the smart farm research center of Gyeongsang National University, Jinju-si, South Korea; the coordinates of the experiment sites are 35°09′06.26″ N, 128°05′043.838″ E [1]. Gyeongsang National University’s Institutional Animal Care and Use Committee (GNU-150508-R0029) approved the experimental procedure. Two identical pig barns were selected with dimensions of 5.4 m length × 3.3 m width × 2.9 m height with a 13.26 m^2^ (1.32 m^2^/pig) polypropylene copolymer slatted floor. The experimental site and sensor placements are shown in Figure 1. Throughout this experiment, ten American Yorkshire × Duroc crossbred pigs were housed in model pig buildings. At the age of sixty days, the initial body mass of these pigs was 31.46 ± 0.86 kg, 32.64 ± 0.81 kg (mean ± standard deviation). A nutritionally balanced dry feed containing 3500 kcal/kg apparent digestible energy (DE) was provided to the pigs twice daily at 09:00 h and 17:00 h. The dry feed for the pigs was rationed 1.5–3.2 kg per day per pig, following the guidelines set forth by the Institutional Animal Care and Use Committee (IACUC) of Gyeongsang National University throughout the experiment. A continuously ventilated fan (Auto-Damper 250, Sanison Co., Ltd., Incheon, Republic of Korea) was installed at a vertical distance of 1.45 m from the ground level to promote air circulation. The average flow velocity of the air was measured to be 0.17 m/s. Due to the absence of a universally accepted benchmark for inexpensive sensors, two adjacent sites were selected for the experiment to authenticate the sensor data. Subsequently, the outcomes were compared, and a significant examination was carried out to verify the effectiveness of the sensor. Two air quality monitoring prototypes were constructed and positioned at a height of 2.8 m in the model pig barns to carry out a co-location test.

## 3. The System Design

### 3.1. System Architecture

**Figure 2:**
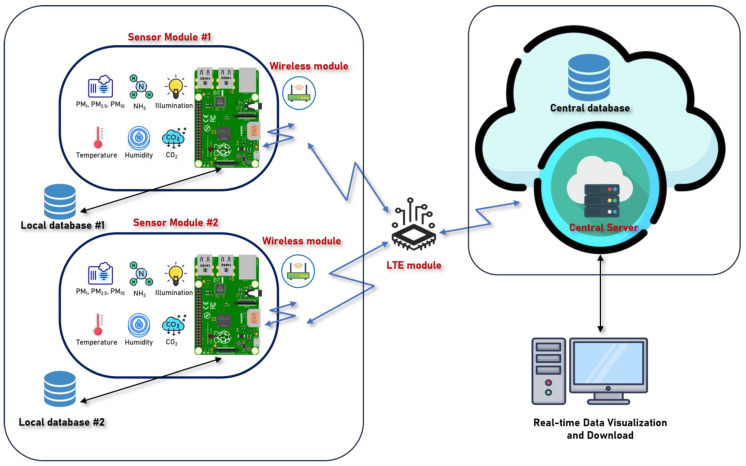
The system architecture for the livestock air quality monitoring system.

The current research framework comprises four distinct layers: the data acquisition layer, the data processing and communication layer, the data storage layer, and the application layer. The study’s framework is depicted in Figure 2. The functions of each layer are as follows:

**Data Acquisition Layer:** The data acquisition layer is primarily responsible for sensing and collecting the desired environmental variables in this air quality monitoring system. Due to the open source nature of the sensor codes, users can readily reprogram the sensors according to their specific needs and thoroughly examine and validate the sensors without encountering any complexities. The Raspberry Pi establishes connections with multiple sensor modules in each sensing node to gather data from the surrounding environment. The Raspberry Pi with Enviro+ board monitors temperature, pressure, humidity, and light intensity. Furthermore, the present iteration of the sensor unit comprises a PMS5003 PM sensor capable of detecting PM_1_, PM_2.5_, and PM_10_ particles and an MH-Z19C sensor designed for detecting CO_2_. Additional information regarding the hardware components and the corresponding hardware cost can be found in the subsequent section.

**Data Processing and Communication Layer:** Data integration between the communication layer and different sensor units occurs in this layer. Data processing and communication were handled by a Raspberry Pi, which is used as a Wi-Fi module to create an access point. This feature facilitates seamless data transmission from the Raspberry Pi to the data storage layer. Various data transmission protocols were generally taken into consideration for data transmission. Hypertext Transfer Protocol Secure (HTTPS) is commonly considered the data transmission method for such IoT frameworks [14,22]. Due to its secure nature and ability to support external encryption and decryption mechanisms. So, the current study also adopts HTTPS for data transmission in the system. No external encryption and decryption mechanisms have been applied in this study, but that feature could be introduced in future studies.

**The Data Storage Layer:** The data storage layer is tasked with the secure storage of data. The current monitoring system employs MySQL as the central database. The current framework encompasses two distinct categories of data storage systems. The data would be stored on the SD card, which is connected to the sensor node, to prevent data loss in certain unfavorable circumstances, such as a loss of Internet connection or a sudden drop in the connection between the server and the sensor gateway. Nevertheless, this approach may not be the most efficient means of data storage despite its inherent value in preserving the data. Upon receiving a request from the central server, the sensor gateway will respond by transmitting the data through the RESTapi obtained from the sensor note by the given query. This method is a dependable approach for data preservation.

**Application Layer:** The application layer is tasked with visualizing the data derived from the web page. The raw data will be stored in the data storage layer until a dashboard, or a web server requests it. The application layer plays a crucial role in the visualization of raw data using charts or the downloading of data for a specific duration by the user. This particular layer is employed for practical purposes.

### 3.2. Hardware Implementation

**Figure 3:**
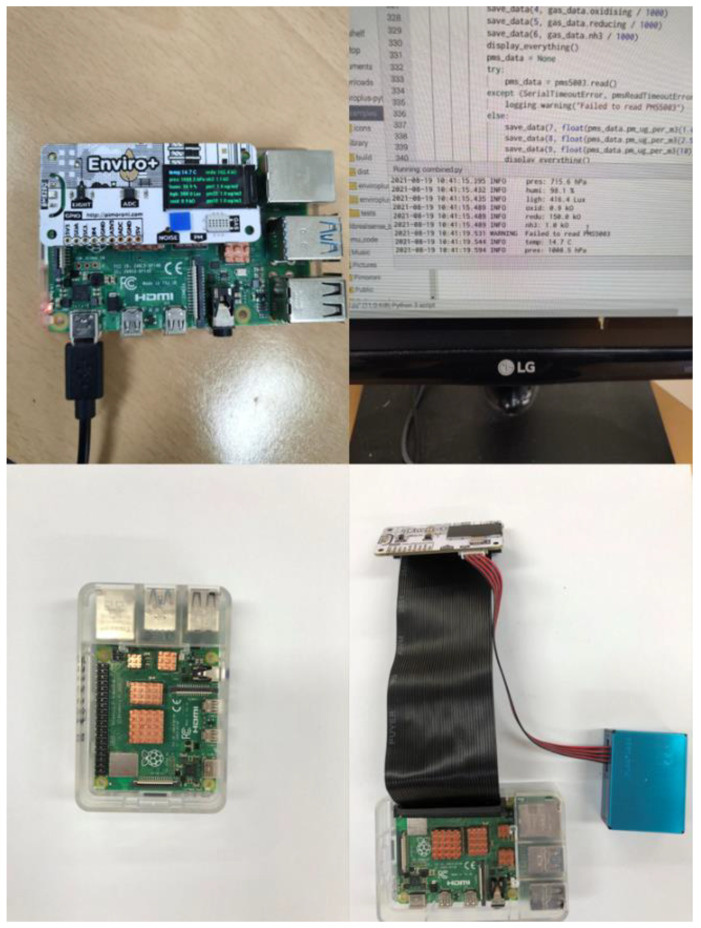
The Raspberry Pi 4 Model B module and it connected to the Enviro + sensor; the sensor sends the signal to the computer during prototype design.

The utilization of a single-board computer (SCB) for the measurement of an indoor microclimate has experienced significant growth in the field of IoT in recent years [13]. The Raspberry Pi is a compact microcomputer that uses either a power source or a battery. It offers several advantages over Arduino boards [23]. Raspbian is an operating system (OS), but it is compatible with several other ARM-Linux OS variants. A Raspberry Pi 4 Model B module was employed as the motherboard for the current air quality monitoring system. This module is a compact and easily configurable SBC (System-on-Chip) that operates on a quad-core Cortex-A72 (ARM v8) Processor with a 64-bit System-on-Chip (SoC) operating at 1.5 GHz. It is designed to consume low power. Furthermore, the device is equipped with 8 GB of LPDDR4 RAM to support the functioning of the Raspbian operating system. The Raspberry Pi module incorporates various functionalities, including a Micro SD port for facilitating external storage access, Bluetooth connectivity, wireless LAN capabilities, USB ports, and GPIO pins for enabling external communication. A Wi-Fi USB adapter was connected to the Gigabit Ethernet port to establish Internet connectivity. A fan and heat sink module were connected to the Raspberry Pi system to disperse surplus operational heat. The current study utilizes the Raspberry Pi 4 Model B module, as depicted in Figure 3.

The current study measured various environmental parameters, including temperature, pressure, humidity, gas levels, light intensity, and particulate matter (PM) concentrations. While there is a wide array of affordable sensors available in the market that are compatible with single-board computers (SBCs), it is crucial to select sensors with high accuracy, reliability, minimal bias, and long lifespans [24,25,26]. The PMS5003 sensor is a robust device capable of effectively monitoring PM_1_, PM_2.5_, and PM_10_ concentrations. Similarly, the MH-Z19C sensor is a low-power, susceptible device that utilizes the non-dispersive infrared (NDIR) principle to detect carbon dioxide (CO_2_) levels. The air quality monitoring system also incorporates an additional sensor array called Enviro+, manufactured by Pimoroni in the United States. Enviro+ is known for its exceptional efficiency due to its compact design, seamless sensor integration, and compatibility with SBCs like Raspberry Pi. Its versatility makes it an ideal choice for research purposes [27,28,29,30]. Raspberry Pi can accommodate additional sensors based on user requirements, thanks to its multiple general-purpose input–output (GPIO) ports. This study uses a Raspberry Pi to communicate with the sensors via these GPIO ports. Enviro+ has dimensions of 65 mm × 30 mm × 8.5 mm and features a 0.96” color LCD that displays data at a resolution of 160 × 80, as shown in Figure 3. It is equipped with a BME280 sensor for measuring temperature, pressure, and humidity, and the LTR-559ALS-01 sensor for measuring light and proximity. The BME280 sensor measures 2.5 mm × 2.5 mm × 0.93 mm and operates on a power supply ranging from 1.71 V to 3.6 V. The LTR-559ALS-01 is an optical sensor capable of measuring illuminance (lux) from 0.01 lux to 64k lux, operating on a voltage supply ranging from 2.4 V to 3.6 V. Both sensors can operate within a temperature range of −30 °C to +70 °C. The PM concentration measurement was performed by connecting a Particulate Matter Sensor (PMS5003) to the Enviro+ device via a universal asynchronous receiver–transmitter (UART) serial interface. Additionally, Enviro+ features a sensor called MICS6814 for measuring analog gas levels. However, to convert data from the analog gas sensor MICS6814 into digital format, an analog-to-digital converter is integrated into the Enviro+ system. Table 1 provides the respective costs of each sensor and Raspberry Pi.

### 3.3. Software Implementation

**Figure 4:**
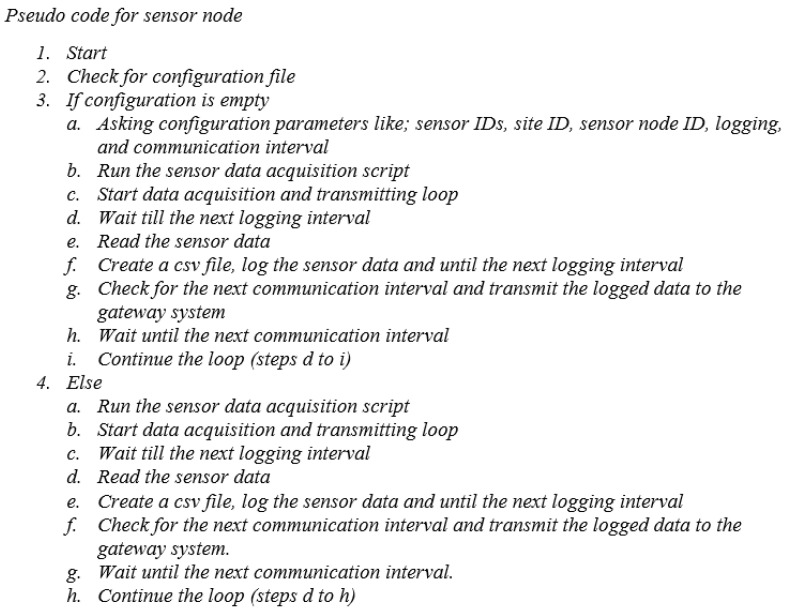
The pseudo-code for the sensor node for sensing data for the air quality monitoring sensor.

**Figure 5:**
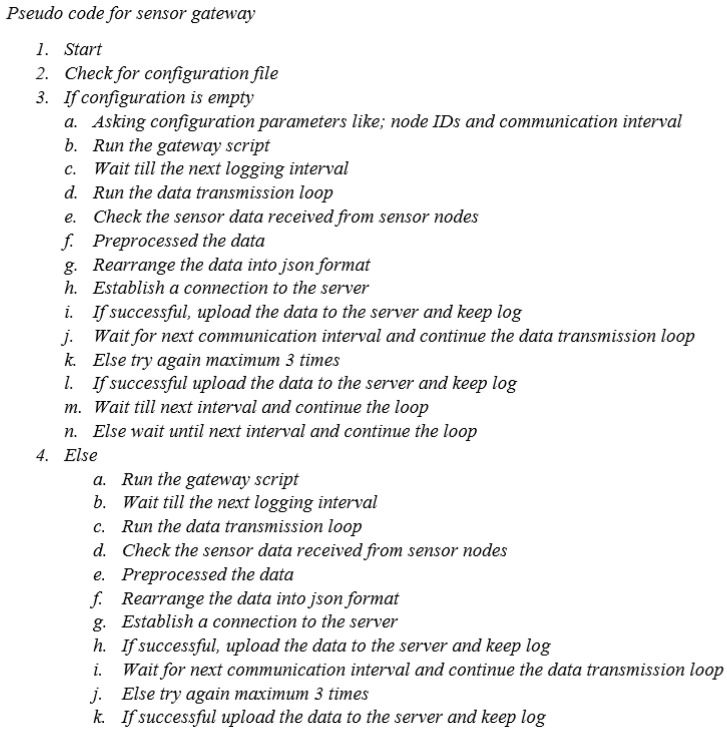
The pseudo-code for the sensor gateway while transferring data from the sensor node to the central server for the air quality monitoring sensor.

**Figure 6:**
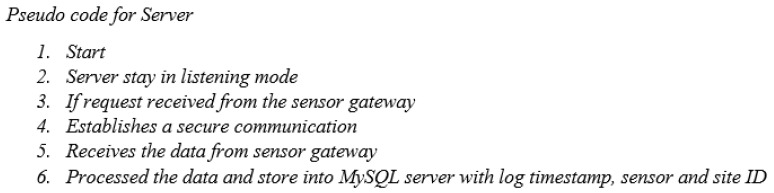
The pseudo-code for the central server while storing the data for the air quality monitoring sensor.

The air quality monitoring system employed in the present study utilizes a Raspberry Pi for sensor communication and manages various tasks such as network establishment, data transmission, and storage on an SD card. To provide a better understanding, the pseudocode for the sensor node’s data-sensing process in the air quality monitoring sensor is shown in Figure 4. Similarly, the pseudocode for the sensor gateway, responsible for transferring data from the sensor node to the central server for the air quality monitoring sensor, is shown in Figure 5. Likewise, the pseudocode for the central server, which is responsible for storing data for the air quality monitoring sensor, is shown in Figure 6. Due to its web-based design, the central server system is configured. All the sensors were configured using the Python library on the Raspbian operating system. PHP serves as both the server language and the platform for developing the graphical user interface (GUI). JQuery and Chart.js were utilized to create data visualizations.

Upon powering on, the Raspberry Pi located at the sensor node will verify the configuration of the sensors. In a connection issue, the sensor data acquisition script will be initiated to restore sensor communications. After establishing successful sensor communication, the system will gather sensor information and store it in the form of a CSV file. This process will be repeated once the subsequent communication interval is reached. Similarly, the sensor gateway will verify the presence of the server and initiate the transmission loop. If the server is verified, an HTTP connection will be established, enabling the transmission of measured data to the MySQL server via the REST API in JSON format. After receiving acknowledgment from the server, the connection will be terminated. The LCD display exhibits numerical data obtained from the sensors. Both online and offline modes facilitate continuous data sensing. Additionally, this technology proves advantageous when environmental monitoring is required in remote areas lacking Internet connectivity. The present iteration of the prototype collects data at intervals of 1 min and enters a state of dormancy following each measurement.

## 4. Results

### 4.1. Co-Location Test

As mentioned earlier, there is no guarantee that a sensor with a low cost will produce accurate data. We implemented two sensor systems in two neighboring buildings to assess their performance. The experimental study was conducted between 1 October 2021 and 1 November 2021. Data were collected at 1 min intervals, resulting in 46,030 data points collected from each pig barn. The data collected exhibit high similarity to those of both pig barns. The descriptive statistics of collected variables from Pig Barn 1 and Pig Barn 2 using the air quality monitoring system are shown in Table 2.

**Figure 7:**
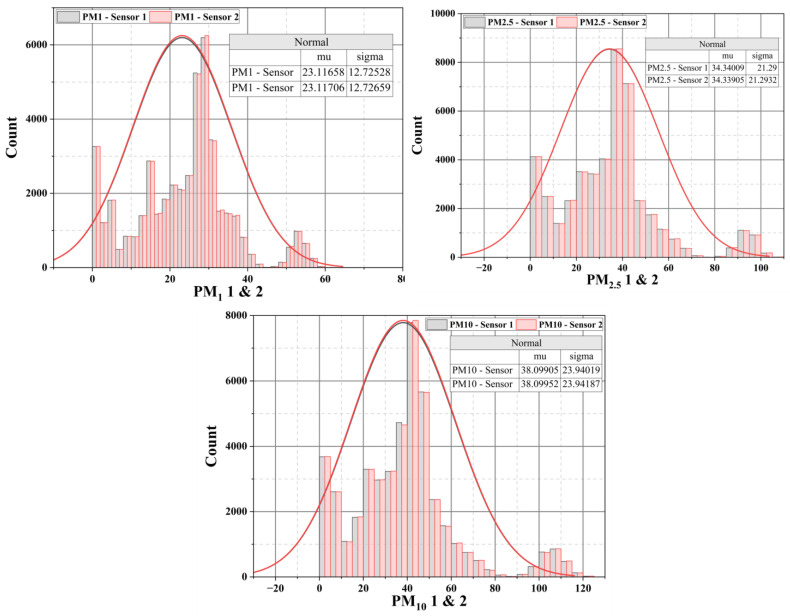
The distribution plots of PM_1_, PM_2.5_, and PM_10_ between Pig Barn 1 and Pig Barn 2.

A thorough examination of both datasets was undertaken to establish the comparability between Pig Barn 1 and Pig Barn 2. In addition to calculating the mean and standard deviation, the current study also considers the minimum value, 25% of the data, the median, 75%, and the maximum value to identify any differences. Except for the disparity in the maximum value of CO_2_ value (Pig Barn 1: 1760; Pig Barn 2: 3618) and PM_10_ levels (Pig Barn 1: 125, Pig Barn 2: 127), all other factors exhibit a high degree of similarity. To mitigate any potential disparities in similarity, the present study examines the distribution of all variables and the mean (mu) and standard deviation (sigma). However, neither site exhibits any disparities in the distribution graph. The distribution plots between the two sites are depicted in Figure 7. This graph explicitly represents the PM_1_, PM_2.5_, and PM_10_ concentrations, as the study primarily focuses on collecting air quality data. Furthermore, in this study, the Wilcoxon Rank Sum Test was employed to assess the independence of the datasets, ascertain equal variance, and examine the distribution of both datasets. Table 3 displays the results, indicating no significant changes were observed among the datasets. Hence, the air quality monitoring system possesses a robust data collection capability, ensuring consistent and reliable results regardless of location.

### 4.2. System Performance

**Figure 8:**
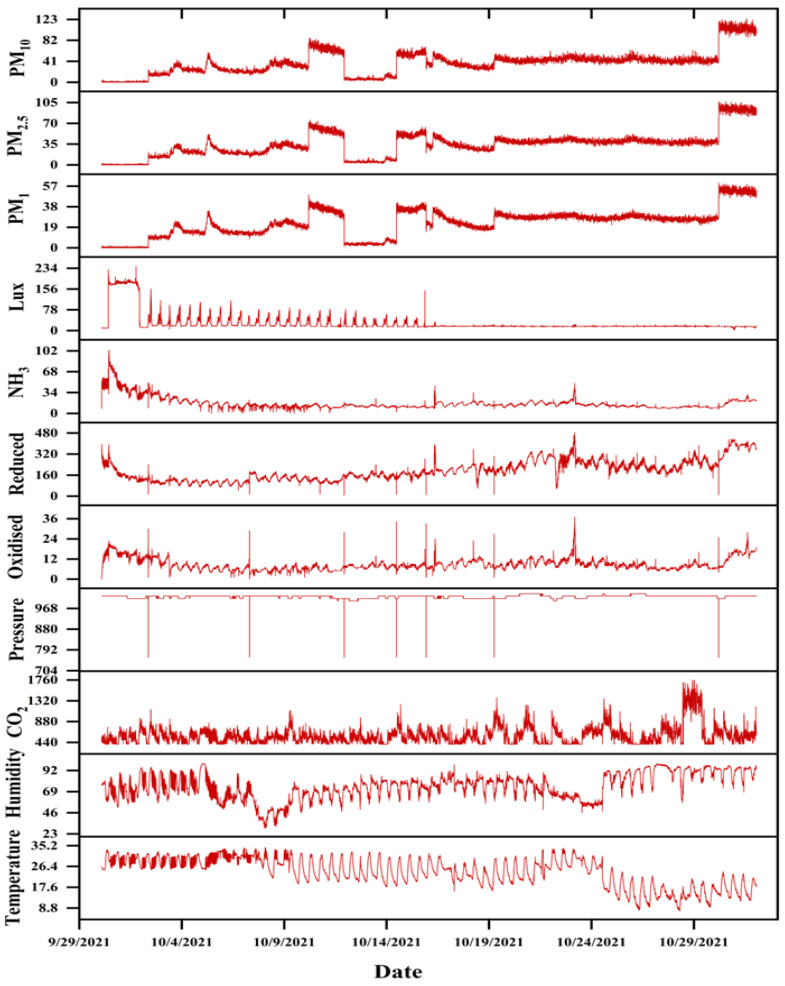
Line plots of all variable data obtained from Pig Barn 1.

**Figure 9:**
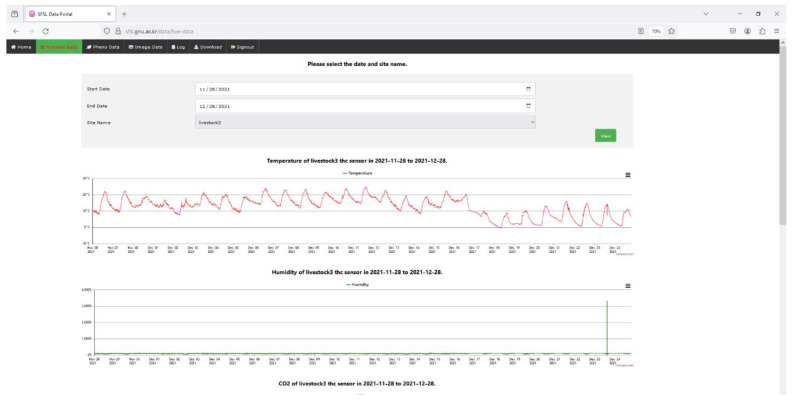
Screenshot showing the graphical user interface (GUI) of the current study and plots showing visualization of variables.

**Figure 10:**
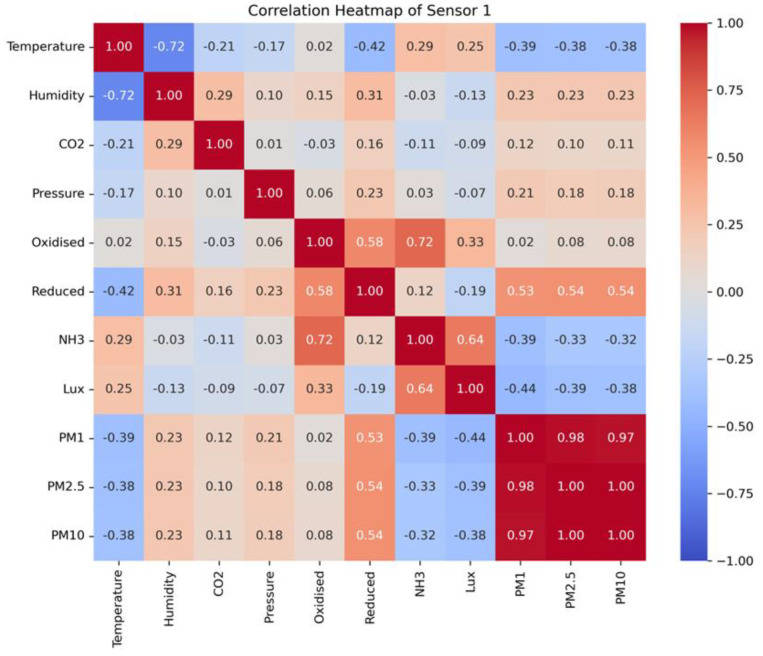
Correlation heatmap between all variables obtained from Pig Barn 1.

Throughout the experiment, our system exhibited enhanced stability and optimal functionality. Establishing an Apache server on a desktop with Microsoft Windows 10 as the operating system is necessary due to the global mode operation of the central web system. However, the central database is constructed using MySQL. Additionally, the server system undergoes daily monitoring to ensure the absence of sudden system failures or data loss throughout the experiment. Figure 8 displays the line plots of all the variables collected throughout the experiment. Given the close resemblance between the Pig Barn 1 and the Pig Barn 2 datasets, the Pig Barn 1 data are selected for the graphs. Similarly, the reliable functioning of sensor nodes depends upon the effective thermal management within sensor nodes. In addition, the prototype was equipped with a cooling fan to avoid heat events. Significant fluctuations in the sensor node’s performance result in the presence of outlier data. Figure 8 additionally guarantees the absence of any outlier data. The graphs depicting the data collected from the two pig barns and stored in the central server are presented through a web-based graphical user interface (GUI). By appropriately selecting the control options, it is possible to present the graph representation of the collected data on the website (sfsl.gnu.ac.kr). Figure 9 demonstrates that the system can display data graphs for all the sensors of a sensing node on a single page. Nevertheless, the authors analyzed the data to determine the correlation between indoor microclimatic variables and PM concentrations. The researchers employed Spearman rank-order analysis to assess the degree of correlation between two measured parameters. This was achieved by assigning a ranked value (R) ranging from −1 to 1 [31]. A heat correlation map has been generated based on the collected variables, as depicted in Figure 10. However, except for reduced (CO), none of the variables exhibit an R-value exceeding 0.5. Nevertheless, PM may positively correlate with various physical parameters, including fan speed, human interaction, airflow, ventilation rate, etc.

## 5. Discussion

### 5.1. Limitations and Obstacles

Several studies have already developed air quality monitoring systems in recent days, yet it is for outdoor or living houses or small buildings. That kind of system also can be adopted for the swine building, but the building dynamics of humans and animal shelters are completely different. The present study demonstrated a low-cost air quality monitoring system and validated it in a real pig barn. However, the authors highlight the practical technical obstacles that arise while implementing such systems and the corresponding methodologies for finding solutions.

**Hardware Limitations:** Hardware durability in indoor air quality monitoring systems can fluctuate based on environmental conditions, usage patterns, technological advancements, and component quality, among other variables. The components of superior-quality hardware typically have longer lifespans than those of inferior quality. The current investigation purchases superior hardware to develop the present monitoring system. For example, while most studies employed Raspberry Pi Zero to fabricate the sensor nodes, the current investigation utilized Raspberry Pi 4B, an even more powerful model capable of supporting greater sensor loads and being compatible with future sensor additions. For measuring air quality parameters, IoT devices rely on sensors. However, the collected data may contain some errors due to these sensors’ variable precision and dependability. While the calibration and maintenance of sensors are critical, they can present difficulties when applied to real-world conditions. In addition to investing in sensors of standard quality to address this obstacle, the current investigation validated the sensors in real-world situations to ascertain their precision and dependability.

**Interference and Environmental Factors:** IoT sensors could experience interference from various environmental and human sources, including but not limited to dust, humidity, temperature fluctuations, and barn cleaning activities. These environmental factors may impact the performance of sensors, resulting in accurate readings or consistent data. Using appropriate casing, the present study protects the sensor nodes from environmental factors that may interfere with measurements.

**Unexpected Collisions:** IoT devices depend on Internet connectivity to transmit data to central servers or cloud platforms for analysis. Data transmission may be impeded, however, by unreliable or restricted Internet connectivity in specific regions; this may cause delays or data loss. This challenges remote or rural areas characterized by inadequate network infrastructure. Several unexpected run-time crashes transpired throughout the data collection phase. Errors in sensor readings, poor connection, overloaded access, and server unavailability contributed to the issues. To address this difficulty, the authors employ local data processing and storage capabilities to temporarily store data during network outages and retransmit it once connectivity is restored. Furthermore, the sensor gateway guarantees consistent data transmission and restarts the data acquisition system in case of unexpected network collisions. Furthermore, local storage is an additional measure to prevent data loss; the log will be transferred to the central database once the network connection is restored.

**Cost and Scalability:** The financial investment required to implement IoT-based indoor air quality monitoring systems can be substantial at the outset, particularly when considering comprehensive solutions that encompass numerous sites or expansive buildings. Further infrastructure expansion or the need to scale up these systems may necessitate additional hardware, software, and maintenance expenditures. The current study opts for inexpensive yet high-quality equipment to align expenses and minimize initial investment. Compared to other studies, the spending associated with developing this monitoring system is reasonable.

The present study effectively addresses the constraints of IoT in indoor air quality monitoring and maximizes the capacity of these systems to enhance occupant comfort and environmental health by implementing these strategies.

### 5.2. Future Directions

The developed prototype’s collection of sensitive data regarding indoor air quality gives rise to apprehensions regarding data privacy and security. Unauthorized access to this information may expose building occupants to potential dangers or compromise their privacy. It can be difficult to implement robust cybersecurity measures effectively and ensure compliance with data protection regulations, even though such measures are vital. Subsequent lines of inquiry could encompass data-related concerns, including the security and privacy of user data, alongside the assessment of diverse privacy preservation methodologies to safeguard user information. The objective of forthcoming research is to develop robust encryption and authentication mechanisms to safeguard data while they are in transit and at rest.

Despite the completion and experimentation of the proposed prototype, the system is still in the developmental phase, with certain constraints that will be rectified or alleviated in subsequent iterations. Certain sensors necessitate calibration and fine-tuning to produce more precise readings. Although the prototype has undergone calibration, the system must be calibrated in various environmental conditions for more precise results. Given that the study’s primary goal is to develop affordable air quality sensors, the focus is primarily on designing the system. As a result, the system is unable to compare data with the traditional, expensive, and calibrated system. Nevertheless, future studies will assess the performance of the current system in comparison to the conventional calibrated air quality monitoring system. Furthermore, the sensor was only deployed in the pig barns for a limited duration, limiting its ability to capture data across various seasons, as the experiment was exclusively carried out in the model pig barn. Future studies will address these limitations by implementing a year-round air quality monitoring system to understand the seasonal impacts on air quality comprehensively.

## 6. Conclusions

This study presents a Raspberry Pi-based air quality monitoring system that is customized to meet the unique requirements of livestock management at an affordable price. This study makes a scholarly contribution by introducing a novel approach to installing a portable, scalable, and cost-effective IoT data infrastructure to sense indoor environments. The proposed wireless distributed sensing network comprises two prototype sensing nodes and the system’s design. Furthermore, it has undergone comprehensive testing and validation in an actual environment and was assessed through co-locating testing. The Wilcoxon rank sum test demonstrates that there are no significant differences between the two sensor datasets, as all variables have a p-value greater than 0.05. The prototypes can generate data about various parameters, including temperature, humidity, light, CO_2_, pressure, NH_3_, different types of gases, PM_1_, PM_2.5_, and PM_10_. However, except for carbon monoxide (CO), none of the variables exhibit an R-value exceeding 0.5 with PM concentrations. A graphical user interface is incorporated into the system to facilitate data access, visualization, and download and to enable adjustments to the sensing nodes and sensor modules. The study recorded the average values of PM1 (23.116 μg/m^3^), PM2.5 (34.34 μg/m^3^), and PM10 (38.099 μg/m^3^) based on the sensor readings. The experimental results demonstrate the proposed system’s functionality, portability, and scalability.

This study’s primary objective is to develop a low-cost air quality monitoring system for pig barns to protect farmworkers, as few are cognizant of the adverse effects of PM concentrations while working in pig barns. PM_2.5_ should not exceed 35 µg/m^3^, as the National Ambient Air Quality Standards for Particle Pollution specified. However, PM_1_, PM_2.5_, and PM_10_ are considerably higher in the table than this standard. Farm owners should consider this type of monitoring system, and it must be repaired. Presently, the system establishes communication with the sensors through the general-purpose input–output ports of the Raspberry Pi. After this, the controlling strategies for the pig barns could be enhanced through the addition of actuators to regulate and improve the air quality within the pig barns. Subsequently, the authors intend to incorporate additional functionalities, such as controls for ventilator fans, to enhance the usability and functionality of the system.

## Figures and Tables

**Table 1:** The expense of the equipment used to make the air quality monitoring system for the pig barn.

S.No	Sensor Name	Detectable Parameter	Price in USD
1	Raspberry Pi 4 Model B	NA	35
2	MH-Z19C	CO_2_	10.64
3	Enviro+	Temperature, pressure, humidity, lux	58.69
4	PMS5003	PM_1_, PM_2.5_, PM_10_	

**Table 2:** The descriptive statistics of collected variables from Pig Barn 1 and Pig Barn 2 by using the air quality monitoring system.

		Pig Barn 1							Pig Barn 2						
Variables	Unit	Mean	SD	Min	25%	Median	75%	Max	Mean	SD	Min	25%	Median	75%	Max
Temperature	°C	24.040	6.067	7.6	20.1	25.3	29.3	34.4	24.041	6.067	7.6	20.1	25.3	29.2	34.5
Humidity	%	74.667	13.93	29	65.2	75.8	84.4	99.6	74.668	13.93	29	65.2	75.8	84.4	99.4
CO_2_	Ppm	586.179	187.86	400	455	551	651	1760	586.267	188.44	400	454	552	651	3618
Pressure	Pa	1017.578	6.62	760	1010	1020	1020	1030	1017.598	6.28	760	1010	1020	1020	1030
Nitrogen dioxide	Ppm	8.901	3.51	0	6	8	10	37	8.898	3.51	0	6	8	10	39
Carbon monoxide	Ppm	195.491	77.57	9	132	182	244	484	195.488	77.59	10	132	182	244	475
NH_3_	Ppm	16.591	10.21	0	11	13	18	103	16.591	10.21	0	11	13	18	102
Illuminance	Lux	28.322	35.65	2	16	17	19	241	28.317	35.64	2	16	17	19	250
PM_1_	μg/m^3^	23.116	12.72	0	15	26	30	60	23.117	12.72	0	15	26	30	60
PM_2.5_	μg/m^3^	34.34	21.29	0	21	36	42	107	34.339	21.29	0	21	36	42	107
PM_10_	μg/m^3^	38.099	23.94	0	23	39	47	125	38.099	23.94	0	23	39	47	127

**Table 3:** Wilcoxon rank sum test results between Pig Barn 1 and Pig Barn 2 datasets.

Variables	Statistic	*p*-Value	Significance
Temperature	−0.0119	0.990507	Not significant
Humidity	−0.01304	0.989599	Not significant
CO_2_	0.043688	0.965153	Not significant
Pressure	−0.07444	0.940658	Not significant
Oxidized	0.146776	0.883309	Not significant
Reduced	0.03978	0.968269	Not significant
NH_3_	−0.02805	0.97762	Not significant
Lux	0.232048	0.816501	Not significant
PM_1_	−0.02035	0.983766	Not significant
PM_2.5_	0.006698	0.994656	Not significant
PM_10_	−0.02254	0.982016	Not significant

## Data Availability

The datasets generated during and/or analyzed during the current study are available from the corresponding author upon reasonable request.
